# A person-centred intervention remotely targeting family caregivers’ support needs in the context of allogeneic hematopoietic stem cell transplantation—a feasibility study

**DOI:** 10.1007/s00520-022-07306-w

**Published:** 2022-08-11

**Authors:** Annika M. Kisch, Karin Bergkvist, Sólveig Adalsteinsdóttir, Christel Wendt, Anette Alvariza, Jeanette Winterling

**Affiliations:** 1 Department of Haematology, Oncology and Radiation Physics, Skåne University Hospital, Lund, Sweden; 2 Institute of Health Sciences, Lund University, Lund, Sweden; 3 Department of Neurobiology, Care Sciences and Society, Karolinska Institutet, Stockholm, Sweden; 4 Department of Care Science, Sophiahemmet University, Stockholm, Sweden; 5 Medical Unit CAST, Comprehensive Cancer Centre, Karolinska University Hospital, Stockholm, Sweden; 6 Department of Health Care Sciences, Palliative Research Centre, Marie Cederschiöld University, Stockholm, Sweden; 7 Capio Palliative Care, Dalen hospital, Stockholm, Sweden; 8 Medical Unit HHLH, Comprehensive Cancer Centre, Karolinska University Hospital, Stockholm, Sweden

**Keywords:** Allogeneic stem cell transplantation, Cancer, Family caregivers, Feasibility, CSNAT-I

## Abstract

**Purpose:**

Allogeneic hematopoietic stem cell transplantation (HSCT) is an intensive curative treatment that increases family caregivers’ burden. The aim of this study was to explore the feasibility of remotely assessing and addressing family caregivers’ support needs in terms of demand and acceptability using the Carer Support Needs Assessment Tool Intervention (CSNAT-I) in the HSCT context.

**Methods:**

CSNAT-I consists of an evidence-based tool and a five-stage person-centred process. The intervention was performed remotely by two designated nurses from two HSCT centres, one before HSCT and the second 6 weeks after (November 2020 to March 2021). To capture the experiences of using CSNAT-I, interviews were conducted with family caregivers and reflections were gathered from the designated nurses.

**Results:**

Of 34 eligible family caregivers, 27 participated, 70% were partners and the rest children, siblings or other relatives. The main support needs were knowing what to expect in the future and dealing with your feelings and worries. The most frequent support actions according to CSNAT-I were psychological support and medical information. Four categories summarised family caregivers and designated nurses’ experiences: CSNAT-I was relevant and became an eye opener; nurses’ experiences were important for enabling trustful CSNAT-I conversations; CSNAT-I provided family caregivers with support and a sense of security; and CSNAT-I gave family caregivers insight and enabled change.

**Conclusion:**

Both family caregivers and designated nurses experienced that using CSNAT-I in an HSCT context was feasible and had the potential to provide valuable support for most of the participating family caregivers.

## Introduction

The rationale behind this study is the lack of feasible person-centred support interventions targeting family caregivers’ (FC) support needs in the context of allogeneic hematopoietic stem cell transplantation (HSCT). HSCT is an intensive curative treatment for hematological malignancies, such as leukaemia, with a high risk of relapse and severe complications for patients, such as graft versus host disease and complex infections [1–3]. The life situation of FC of HSCT patients is also affected and their distress is sometimes even higher than that of the patients [4, 5]. They have to deal with their own worries about living with a seriously ill patient and the threat of death [6], as well as being responsible for physical and psychological support [5]. FC support needs differ between individuals and also between time points during the HSCT process [7]. Their individual characteristics and social context may influence their ability to provide support for the patient, which is often influenced by the patient’s constantly changing health status [8]. Until today, few intervention studies have explored how individual support to FC in the HSCT context can increase their own well-being [9, 10]. However, two interventions in the USA have shown improvements. These interventions included three [9] and eight [10] sessions, respectively, and many FC did not want to participate or dropped out during the intervention due to their own strain during the transplantation. To find a shorter person-centred intervention that can be given to all FC to capture those that need further support might therefore be valuable.

Based on interviews with FC, researchers in palliative care have developed The Carer Support Needs Assessment Tool Intervention (CSNAT-I). The intervention is specifically developed to directly assess and address practical, emotional, existential, and social support needs [11]. Conversation-based assessments enable each FC to identify her/his specific support needs and prioritize the most important ones [12]. The CSNAT-I has two parts: an evidence-based tool and a five-stage person-centred process. The tool has recently been revised and one domain about relationships has been added based on findings from interviews with family caregivers of persons with motor neuron disease [13]. The current version of the tool (v 3) includes 15 domains about the need for more support, reflecting the dual role of FC as both providers of care and persons in need of support. The five-stage process starts with an introduction to the CSNAT-I, followed by time for the FC to reflect upon support needs. In a conversation with one healthcare professional, FC discuss and prioritize their support needs. The conversation results in a shared support plan to address the prioritized support needs, of which some can be directly dealt with during the meeting [11, 12]. The CSNAT-I has been shown to facilitate the assessment of support needs and ensure adequate support [14, 15]. It has been translated into several languages including Swedish [16] and is used internationally in palliative care.

There are both similarities and differences between a palliative care context and the HSCT context. The similarities are the complexity of the illness and associated complications, which require an advanced level of highly specialized care. The differences are that in palliative care the patient’s condition deteriorates and ends in death, while in HSCT care the treatment has a curative intent and patient’s medical and health status often change rapidly. There is a need for feasible person-centred support interventions targeting FC support needs in the context of HSCT. Therefore, the aim of this study was to explore the feasibility of remotely assessing and addressing family caregivers’ support needs in terms of demand and acceptability using the Carer Support Needs Assessment Tool Intervention (CSNAT-I) in the HSCT context.

## Methods

### Design

**Fig. 1:**
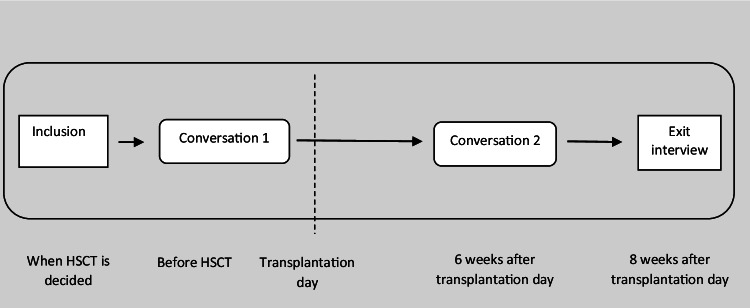
Design of the feasibility study using CSNAT-I in the HSCT context

This feasibility study has a longitudinal design (Fig. 1). The focus is on demand, i.e. how much the intervention is used, and acceptability, i.e. the extent to which those delivering or receiving the intervention find it appropriate, satisfying and attractive [17].

### Sample and procedure

Adult FC were consecutively included from two HSCT centres, namely Stockholm and Lund. Patients were asked by the HSCT coordinator to select a FC involved in their everyday life. With the patients’ agreement, the HSCT coordinator sent the study information to the FC, who were then contacted by telephone, given oral information and invited to participate in the study. If they agreed, they were included in the CSNAT-I. The inclusion period was from November 2020 to March 2021 and the inclusion criteria were being a FC to a patient undergoing HSCT and able to read and speak Swedish.

### The CSNAT-I in the HSCT context

One designated nurse from each HSCT centre performed the CSNAT-I. They had 14 and 23 years of nursing experience respectively, had specialist training in oncology and long experience of HSCT. Both were included in the planning phase of using the CSNAT-I in the HSCT context. Initially, the designated nurses completed training about how to use the CSNAT-I. A Swedish version of the original CSNAT instructions was used, including information and explanations about the domains and the five-stage person-centred approach. Instructions are based on the online toolkit that is available in English. The CSNAT-I conversations were carried out at two time points, before transplantation and 6 weeks later when patients are usually back home again after the intensive inpatient treatment. The decision to select these two time points was based on clinical experience within the research group and earlier studies showing that these time points are demanding for the FC with uncertainty and changes in everyday life including responsibilities that often involve the need for support [7, 8]. For inclusion in the study, the FC had to take part in the two CSNAT-I conversations and the exit-interview. Due to the Covid-19 pandemic, the CSNAT-I had to be delivered remotely by telephone or video-visits. The CSNAT tool was sent by post to the FC before conversation 1.

### Data collection

#### FC support needs and support plan

The Swedish CSNAT tool version 3 and its support plan were used [12, 15, 16]. The tool asks the FC “Do you need more support with…” and includes 15 domains. The response alternatives for each domain are no, a little more and quite a bit more. The support plan includes further actions required to address FC needs. To document FC support needs, the nurses filled in the tool for each FC during conversation 1.

#### Exit interviews with FC

Semi-structured telephone interviews were conducted by two of the authors (AMK, JW) after the participant had completed the second conversation. The interviews evaluated the participants’ experiences of the CSNAT-I, including whether participation had led to any changes and suggestions for improvements. The median duration of these interviews was 29 min (range 16–48). The interviews were recorded and closely followed by a written summary of the answers and field notes were made during and directly after the interviews.

#### Designated nurses’ memos from conversations

After each of the two conversations, the nurses filled in a standardized written documentation form including questions about their overall reflections on the conversation, their experience of using the CSNAT-I and suggestions for improvements.

#### Reflective conversations with the designated nurses

After all conversations were completed, the designated nurses reflected individually on their experiences of the conversations together with one of the authors (KB). These conversations were recorded with a written summary.

### Data analysis

Descriptive statistics were used to describe the characteristics of the participants. Inductive qualitative content analysis was applied to the exit interviews, the reflective conversations with the designated nurses and the memos. The written summaries were read through several times to get a full picture of the complete data [18]. In the next step, open coding from the perspective of acceptability [17] was conducted by two of the authors (AMK, KB). Meaning units and codes were recorded on a coding sheet and finally grouped into categories. The qualitative analyses resulted in four categories. The analyses were continuously discussed between all authors and the categories were adjusted until consensus was achieved. Interpretation to a manifest level was used throughout the analysis process.

### Ethical considerations

In the written and oral information, we emphasised the voluntary nature of participation, the right to withdraw from the study at any time, that data would be treated confidentially and that the identity of the participants would be protected. Ethical approval was obtained from the Regional Ethical Review Board in Stockholm, Sweden (No. 2017/1112–31/4).

## Results

The findings are presented first with data about the participants, and then an overview of the support needs and support actions in the HSCT context. Thereafter, FC and designated nurses’ experiences of the CSNAT-I is presented in four categories: the CSNAT-I was relevant and became an eye opener; nurses’ experiences were of importance to enable trustful CSNAT-I conversations; the CSNAT-I provided FC with support and a sense of security; and the CSNAT-I gave FC insight, preparedness and enabled change. Quotations are presented to illustrate the family caregivers’ and nurses’ experiences in their own words. Out of 50 eligible patients planned for HSCT, 45 underwent transplantation, four were postponed and one died before transplantation. Among those 45 patients, six had no FC who understood Swedish, two did not allow us to invite their FC to participate, one had no FC, in one case the pre-transplantation process was too quick to enable us to ask her/him to participate and in another it was not possible to ask the patient for permission due to cognitive deficits. Thus, 34 FC were eligible for inclusion and informed about the study, of whom four declined participation and 30 accepted (88%). Of the 30 FC who accepted, three only participated in conversation 1, which resulted in a total of 27 (80%) FC, 19 from Stockholm and 8 from Lund. All conversations were conducted over the phone, except for one by video. Conversation 1 took 45–60 min and conversation 2 13–30 min. The median age of the participants was 55 years and 56% were women. Other characteristics are presented in Table 1.

**Table 1:** Characteristics of the participating FC (*n* = 27)

FC age, years, Md [min–max]	55 [22–73]
FC gender, *n* (%)	
Female	15 (56)
Male	12 (44)
FC country of birth, *n* (%)	
Sweden	23 (85)
Elsewhere	4 (15)
FC relationship to patient, *n* (%)	
Partner	19 (70)
Child	4 (15)
Sibling	2 (7)
Other (Cousin, friend)	2 (7)
Cohabiting with the patient, *n* (%)	
Yes	18 (67)
No	9 (33)
Education, *n* (%)	
Lower (elementary or secondary school)	14 (51)
Higher (college/university)	13 (49)
Occupational situation	
Working full-time	11 (41)
At home due to HSCT for more than 4 weeks*	4 (15)
Disability pension/sick leave due to other reason	2 (7)
Old age pension	7 (26)
Other (housewife, jobseeker, student)	3 (11)
Time from patient’s diagnosis to start of the CSNAT-I intervention	
3–5 months	10 (37)
6–11 months	9 (33)
> 12 months (range 17–156 months)	8 (30)

^*^Have been on sick leave or in receipt of a disease carrier allowance due to COVID-19

### Support needs and support actions in the HSCT context

**Fig. 2:**
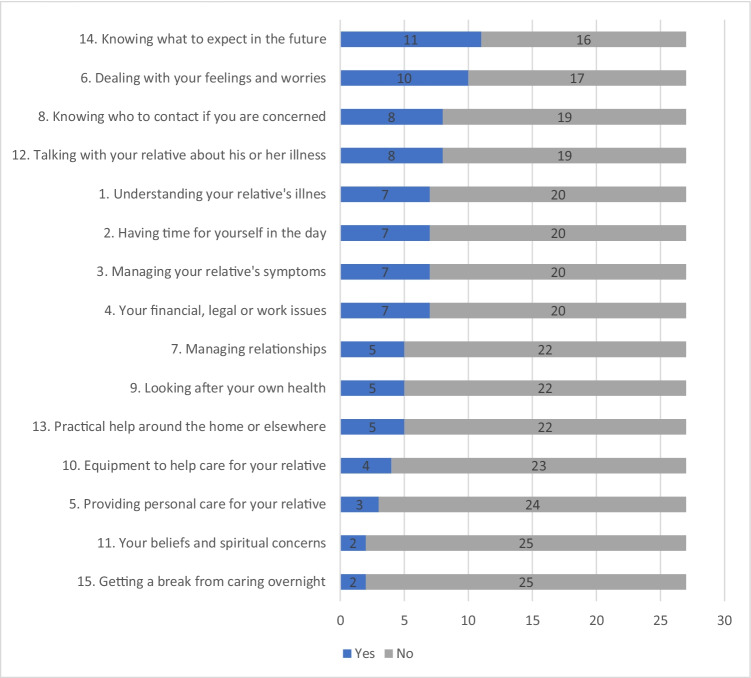
Number of family caregivers expressing more support needs listed in the CSNAT-I during conversation 1 (*n* = 27). The “Yes” is a sum of the response alternatives selected as “a little more” and “quite a bit more”

The number of support needs reported by FC in the CSNAT tool at conversation 1 varied widely: zero (*n* = 4), one (*n* = 6), two (*n* = 4), four (*n* = 4), five (*n* = 2) and more than six (*n* = 7). The most frequent support needs being present in the CSNAT tool were knowing what to expect in the future; dealing with your feelings and worries; knowing who to contact; and talking with your relative about their illness. In Fig. 2, the “Yes” is a sum of the response alternatives selected as “a little more” and “quite a bit more”. A support plan was set up in 78% (21 out of 27) of the first conversations, while in 22% (6 out of 27) there was no need for further action (Table 2). The actions concerned advice about how to seek psychological support (*n* = 14) or medical treatment for themselves (*n* = 2), medical information about the patient from a physician (*n* = 9) or patient organisation (*n* = 4), or how to arrange home care (*n* = 4).

**Table 2:** Number of planned support actions in each of the CSNAT-I domains documented in FC support plan in conversation 1

CSNAT domain	Action plan	*n*
1. Understanding your relative's illness	Talk to the patient’s doctor Participate in the enrolment/discharge talk Use information on patient organisation website Take contact patient organisation for relatives	5 3 3 1
2. Having time for yourself in the day	Continue contact with social worker Referral to the social worker Increase help from other relatives Take a walk outside the home by her own	1 1 1 4
3. Managing you relative’s symptoms	Referral to the social worker Referral to ASIH that can manage patient’s medicines Talk to the patient’s doctor to understand medications	1 1 1
4. Your financial, legal or work issue	Follow-up at the second conversation Referral to the social worker Take contact with own doctor in primary care Take contact with a relative that can help	1 2 1 1
5. Providing personal care for your relative	Take contact with the municipality for home care	1
6. Dealing with your feelings and worries	Continue contact with social worker Referral to the social worker Take contact patient organisation for relatives	1 7 2
7. Managing your relations	Referral to the social worker at ASIH	1
8. Knowing who to contact if you are concerned	Pointed out who at the ward or out-patient clinic that can contact Pointed out that can always call the hospital chaplain	2 1
9. Looking after your own health	Contact with primary care doctor to get an own sick leave Take 10 min for oneself each day Get back to be physically active again with support from physiotherapist Contact with a doctor of naprapathy to get help with own pain	1 1 1 1
10. Equipment to help care for your relative		0
11. Your beliefs and spiritual concerns	Will search for someone else to talk to in addition to social worker Take contact with hospital chaplain	1 1
12. Talking with your relative about his or her illness	Referral to the social worker Take contact patient organisation for relatives Take contact with hospital chaplain Talk to the health care personnel at the ward	5 1 1 1
13. Practical help in the home or elsewhere	Take contact with the municipality for home care Talk to health care personnel at the ward before discharge Engage the family more for practical help	2 1 1
14. Knowing what to expect in the future	Participate in continuous contact with patient’s doctor and nurses Use information on patient organisation website Take contact patient organisation for relatives Participate in the enrolment/discharge talk Take contact patient organisation for relatives Referral to the social worker Give information about normal procedure and side-effects	3 1 2 3 1 2 2
15. Getting a break from caring overnight		0

### FC and designated nurses’ experiences of the CSNAT-I

#### The CSNAT-I was relevant and became an eye opener

Both FC and designated nurses described that using the CSNAT tool provided a structure, which facilitated a good conversation. The designated nurses experienced that the FC were well prepared and believed it had worked well both when FC had completed the tool before the conversation or together with them during the conversation. Many FC highlighted that going through all domains provided them with a new insight into what support they might need throughout the HSCT-process, which was described as “a kind of eye opener” and as a recognition of what might arise in the future.

The domains in the tool were relevant, although some FC, especially those not living with the patient, stated that the domains “Getting a break from caring overnight” and “Practical help in the home” were not relevant. This was also supported by the nurses. Some of the FC commented that the domain “Beliefs or spiritual concerns” felt odd. Only a few of the FC felt the domains were not relevant and some FC appreciated an explanatory description of the domains in relation to the HSCT-process. The FC who found the domains not relevant were mostly FC who were not the partner of the patient or not living together with the patient.

Most of the FC experienced the conversations as “timely” which was confirmed by the nurses. Most FC mentioned that they were content with one follow-up conversation, but a few FC would have appreciated an additional follow-up conversation, which the nurses also identified in a few cases. The nurses reflected on the fact that the content of the conversations had a different focus, i.e. the first conversation often had a focus on “Understanding the illness”, “Expectations for the future” and “Knowing who to contact”. In the second conversation, the support needs identified during the first conversation were followed up and the focus was on dealing with practicalities, such as coping with various restrictions. The nurses experienced that the FC took the lead in the second conversation.

“Good to start from the domain and take them one at a time, very important because then I started to understand what it was all about with everything around us. We had received a lot of information from the doctors and nurses before and therefore I did not think I needed this, but these conversations filled another need.” (Husband, 56 years).

#### Nurses’ experiences were of importance to enable trustful CSNAT-I conversations

All FC identified several factors of importance for creating trust in the nurses, i.e. the nurses’ high level of competence and extensive experience of HSCT, their ability to have these conversations, as well as their professional and personal qualities. The nurses were described as being easy to talk to and being professional yet personal. The designated nurses also highlighted the fact that their experience and knowledge of HSCT-nursing made them comfortable and confident to have these conversations. They emphasised the need to have a genuine interest in listening. They expressed that an active listening approach had developed from their knowledge, experience and conscious choice of a humble attitude towards the life situation of FC.

Both FC and designated nurses reported that conducting the conversations over the phone was positive, worked well and enabled them to build a trustful relationship despite being unable to see each other. Several FC mentioned the advantage of not having to go to the hospital. However, those who had difficulties with the Swedish language would have preferred face-to-face conversations and several FC expressed a wish for video-based conversations. The designated nurses reported a disadvantage of being unable to read body language or interpret reactions and emotions during the conversations. This was especially problematic when a FC cried or shared something sensitive, as it was impossible to show care and provide comfort through their own body language.

“You need to have experience, have worked with patients and know about treatments, how the patient will feel in the future, follow-up etc. Answering the CSNAT tool will give them a sense of security…, used to talking to people, listening, maybe not coming up with solutions so quickly, but asking what do you think, what do you want to do?” (Designated nurse).

#### The CSNAT-I provided FC with support and a sense of security

All the FC expressed and appreciated that the conversations had focused on their life situation and needs. The conversations were about how the FC was doing, her/his thoughts and worries. Many of the FC expressed that they had initially believed that they did not need support, but that during the conversations it became obvious that they felt worse than they had thought. They experienced that the conversations were supportive and derived great benefit from them. The FC expressed that the conversations fulfilled another function compared to the information from doctors, i.e. focus on them as individuals. The FC felt relieved and more at peace after the conversations. For many, this was the first time that focus was exclusively on their life situation and needs, which they expressed as valuable and a good feeling, adding that they appreciated being listened to. The conversations gave them opportunities to ask questions, receive information and reflect and discuss situations in life, with a focus on their needs as a FC, while the patient was not present. The support from the nurses during the conversations also included practical aspects and advice, such as information about support available for FC, for example sick leave, and what to think about during the rehabilitation when the patient returned home. However, some of the FC expressed that they had no need for these conversations. Nevertheless, both designated nurses felt that most of the FC were satisfied after the conversations. Even the FC who did not have any support needs expressed that the conversations per se had been rewarding.

“Very valuable to talk to and get support from someone with expertise in illness and treatments, without medical focus, and with warmth with a focus on me as a relative.” (Wife, 66 years).

#### The CSNAT-I gave FC insight, preparedness and enabled change

When the FC recalled the CSNAT-I during the exit-interviews, they realised that they had gained new insights. They had learned about how they were actually feeling and what support they needed, e.g. the need to take care of oneself and ask for help. They had also gained new knowledge about the transplantation process and the patient’s situation, which led to reduced or more balanced worries about things that might happen later. The intervention also made it possible for them to focus on non-medical aspects, such as how to relate to the situation and that they as FC are important for the patient. They described feeling more prepared for the transplantation process with a sense of participating in the patient’s care. The feeling of preparedness mainly concerned being mentally prepared. For some, the CSNAT-I had led to a personal change in their life situation, and they understood that they needed help. These changes involved dealing with grief, contacting a social worker for psychological support and prioritizing their own needs, such as time for themselves, physical exercise and sufficient sleep during the night. However, a few did not make any changes after the conversations, despite the fact that most appreciated the conversations focusing on them as FC.

“I got an insight into what I needed. I need and it’s okay that I’m sad and grieving and that I need support, I also gained insight into how I can be an even better support for him. It is important that I understand that I have to take care of myself as well because it then helps him. I did not realize that until then, you cannot just move on forward. It was the big win to get this win. Plus, how I handle, got better at handling my grief.” (Friend, male, 59 years).

## Discussion

This feasibility study conducted in an HSCT context shows that the demand from FC to participate in CSNAT-I was high. Furthermore, it demonstrated a high acceptability both among designated nurses and participating FC. Participants described not only appreciating the conversations, but also being provided with support and a sense of security. Several FC experienced gaining insights, preparedness and in some cases the intervention enabled them to make changes in their life. In summary, implementing the CSNAT-I in HSCT general practice is clearly acceptable.

Almost 80% of eligible FC participated in the intervention. Including FC in interventions during the patient’s transplantation process [19] has been previously reported as challenging. However, we believe that our design facilitated FC participation, i.e. the nurses were flexible concerning the time points for the conversations, the conversations were conducted remotely and FC were not asked to fill in any further questionnaires. Most FC were partners, but it was interesting to note that not all patients chose their partner to be the FC in the study. This was probably because they did not want to burden their partner any further and in some cases the partner was also ill and unable to be a FC.

In the present study, all FC and designated nurses were satisfied with the CSNAT-I, including the remotely conducted conversations and the chosen time points for them. The FC expressed satisfaction with the conversations, according to given circumstances, and how they were conducted, i.e. by telphone, the support given by the nurses, with focus on the situation of the FC. However, the FC and the nurses reflected on possible improvements in the intervention, e.g. with converstions face-to-face it would be possible to read and show body language. In general, the first conversation was longer than the second one, which is in line with the developers’ intention [14, 15], as the first includes assessment, delivery of support and documentation, while the second is a follow-up. The results highlight the fact that the CSNAT tool works as a conversation-based assessment between FC and healthcare professionals in an HSCT-context. The person-centred process gave FC the opportunity to gain new insights and receive proactive support. Many participants described that they realised during the first conversation that the patient’s health could deteriorate during and after the HSCT, which increased their preparedness. This finding is very positive, as our earlier study shows that preparedness does not decrease over time among FC in the HSCT context [20]. The insight gained by FC in the present study was mainly related to two of the domains in the CSNAT tool, namely caring for the patient during the night and giving practical help to the patient at home. It is interesting to note that these were the domains that both FC and designated nurses found irrelevant in the first conversation. This was especially reported by FC who did not live with the patient and was not expecting to be involved in such care. However, all domains in the tool were used in an HSCT context by some of the FC both in this and in our earlier study [7], indicating that FC have support needs in all domains.

The results show that the designated nurses’ extensive experience of HSCT care and their ability to provide psychosocial support was highly appreciated by FC. The importance of the abilities of the healthcare professional conducting the conversations is also highlighted in another CSNAT-I feasibility study among FC of patients with chronic obstructive pulmonary disease, who stated that the interventionists’ demeanor, relational skills and knowledge were more important than her/his profession [21]. In the present study, the nurses’ medical knowledge and experience seem to be important to FC, indicating that nurses are an appropriate professional category to conduct the CSNAT-I in HSCT care. Furthermore, we believe that after adequate training the CSNAT-I can also be used to enable more novice nurses to perform this intervention as intended.

In our context, FC described that participating in the CSNAT-I gave them a sense of security. The support plans of more than half of the FC included a need for more psychological support for themselves. This is related to the fact that in the HSCT context the greatest problem for FC is the sense of uncertainty, especially due to the unknown prognosis of the transplantation [6] and that lack of information, incomplete understanding of the treatment and disease, as well as the difficulties coping with the precariousness of daily life increase such uncertainty [22–24]. Furthermore, the need to prioritize themselves including seeking psychosocial support was described by FC as an eye opener, which is also reported in other feasibility studies using the CSNAT-I [25, 26]. Although FC have a personal responsibility to ask for and receive support, we believe that support resources could be offered in connection with the CSNAT-I conversation.

A strength of this feasibility study is that it was based on quite a large sample, was undertaken in two HSCT centres at two different geographical locations and that the two designated nurses were involved in the planning process of delivering the CSNAT-I in the HSCT context. However, having such dedicated nurses delivering the intervention makes it unique and a possible limitation is that the nurses might have been evaluated rather than the effects of the intervention itself. All the authors have vast experience in haematology, oncology and HSCT care, and one of the authors (AA) has high competence in palliative care. However, none of the authors were involved in the care of the recipients related to the family caregivers in the study. Although the authors’ pre-understanding could have influenced the data analyses and interpretation of the data, awareness of this risk and the continuous dialogue between the authors helped to minimize potential misinterpretations.

In conclusion, both FC and designated nurses experienced that using CSNAT-I in the HSCT context was feasible. The intervention had the potential to provide most of the FC with both direct and proactive support. This study also highlights a need for further psychosocial support among FC.

## Data Availability

Not applicable.
